# Insulin-like growth factor-1 level is a poor diagnostic indicator of growth hormone deficiency

**DOI:** 10.1038/s41598-021-95632-0

**Published:** 2021-08-09

**Authors:** Hideyuki Iwayama, Sachiko Kitagawa, Jyun Sada, Ryosuke Miyamoto, Tomohito Hayakawa, Yoshiyuki Kuroyanagi, Taichiro Muto, Hirokazu Kurahashi, Wataru Ohashi, Junko Takagi, Akihisa Okumura

**Affiliations:** 1 Department of Paediatrics, Aichi Medical University, Nagakute, Aichi Japan; 2 Department of Paediatrics, Daiyukai General Hospital, Ichinomiya, Aichi Japan; 3 Division of Biostatistics, Clinical Research Center, Aichi Medical University, Nagakute, Japan; 4 Division of Endocrinology and Metabolism, Department of Internal Medicine, Aichi Medical University, Nagakute, Aichi Japan

**Keywords:** Biomarkers, Endocrinology

## Abstract

We evaluated the diagnostic accuracy of insulin-like growth factor-1 (IGF-1) for screening growth hormone deficiency (GHD) to determine the usefulness of IGF-1 as a screening test. Among 298 consecutive children who had short stature or decreased height velocity, we measured IGF-1 levels and performed growth hormone (GH) secretion test using clonidine, arginine, and, in cases with different results of the two tests, L-dopa. Patients with congenital abnormalities were excluded. GHD was defined as peak GH ≤ 6.0 ng/mL in the two tests. We identified 60 and 238 patients with and without GHD, respectively. The mean IGF-1 standard deviation (SD) was not significantly different between the GHD and non-GHD groups (p = 0.23). Receiver operating characteristic curve analysis demonstrated the best diagnostic accuracy at an IGF-1 cutoff of − 1.493 SD, with 0.685 sensitivity, 0.417 specificity, 0.25 positive and 0.823 negative predictive values, and 0.517 area under the curve. Correlation analysis revealed that none of the items of patients’ characteristics increased the diagnostic power of IGF-1. IGF-1 level had poor diagnostic accuracy as a screening test for GHD. Therefore, IGF-1 should not be used alone for GHD screening. A predictive biomarker for GHD should be developed in the future.

## Introduction

Growth hormone (GH) secretion needs to be assessed for the diagnosis of GH deficiency (GHD) by stimulation tests. However, there are several challenges associated with the GH secretion test^1^. Pharmacological stimuli are not physiological, and their accuracy is poor. It is well known that normally growing children may have falsely low GH responses. Moreover, the diagnostic criteria for GHD are not uniform worldwide^2^. Furthermore, the GH secretion test may be influenced by factors such obesity, undernutrition, sex, age, puberty, and presence of chronic diseases. It also has potential adverse reactions and may sometimes result in hospitalization. Therefore, a predictive biomarker for GHD is desired to avoid unnecessary GH secretion test.

Insulin-like growth factor-1 (IGF-1) is a small polypeptide hormone secreted by the liver when stimulated by GH. As serum levels of IGF-1 show little circadian variation, IGF-1 has been considered as a predictive biomarker for GHD^2^. The utility of IGF-1 for the screening of GHD was reported in some studies^3–7^ but not in others^1,8^. As the study settings in these reports were different, it is difficult to compare the diagnostic accuracy of IGF-1. For example, the inclusion criteria for GH secretion test comprise not only short stature but also bone age^2^, target height^3,6^, and catch-up growth^3^. Furthermore, the studies used different GH cutoff levels^1,3–6,8,9^. Therefore, a prospective cohort study was required to determine the diagnostic accuracy of IGF-1. We prospectively analyzed a cohort of children with short stature to evaluate the diagnostic accuracy of IGF-1 for the diagnosis of GHD.

## Materials and methods

### Patients

This was a prospective cross-sectional study on children with short stature or decreased growth velocity who were examined at Aichi Medical University Hospital between April 2015 and March 2020. All evaluations and procedures were performed in accordance with the Declaration of Helsinki and the Ethical Guidelines for Medical and Health Research Involving Human Subjects established by the Japanese Government. We used the following inclusion criteria: (a) referred to Aichi Medical University for the evaluation of short stature or decreased growth velocity; (b) short stature of ≤  − 2 standard deviation (SD) or height velocity of ≤  − 1.5 SD in > 2 years below the mean for sex and age^10^; and (c) > 1 year of age and before the completion of puberty, according to Tanner stages. The exclusion criteria were the presence of recognized congenital abnormalities, such as hypothyroidism; small for gestational age; Turner’s syndrome; and trisomy 21. The patients who received GH or IGF-1 treatment prior to the study were also excluded.

**Figure 1:**
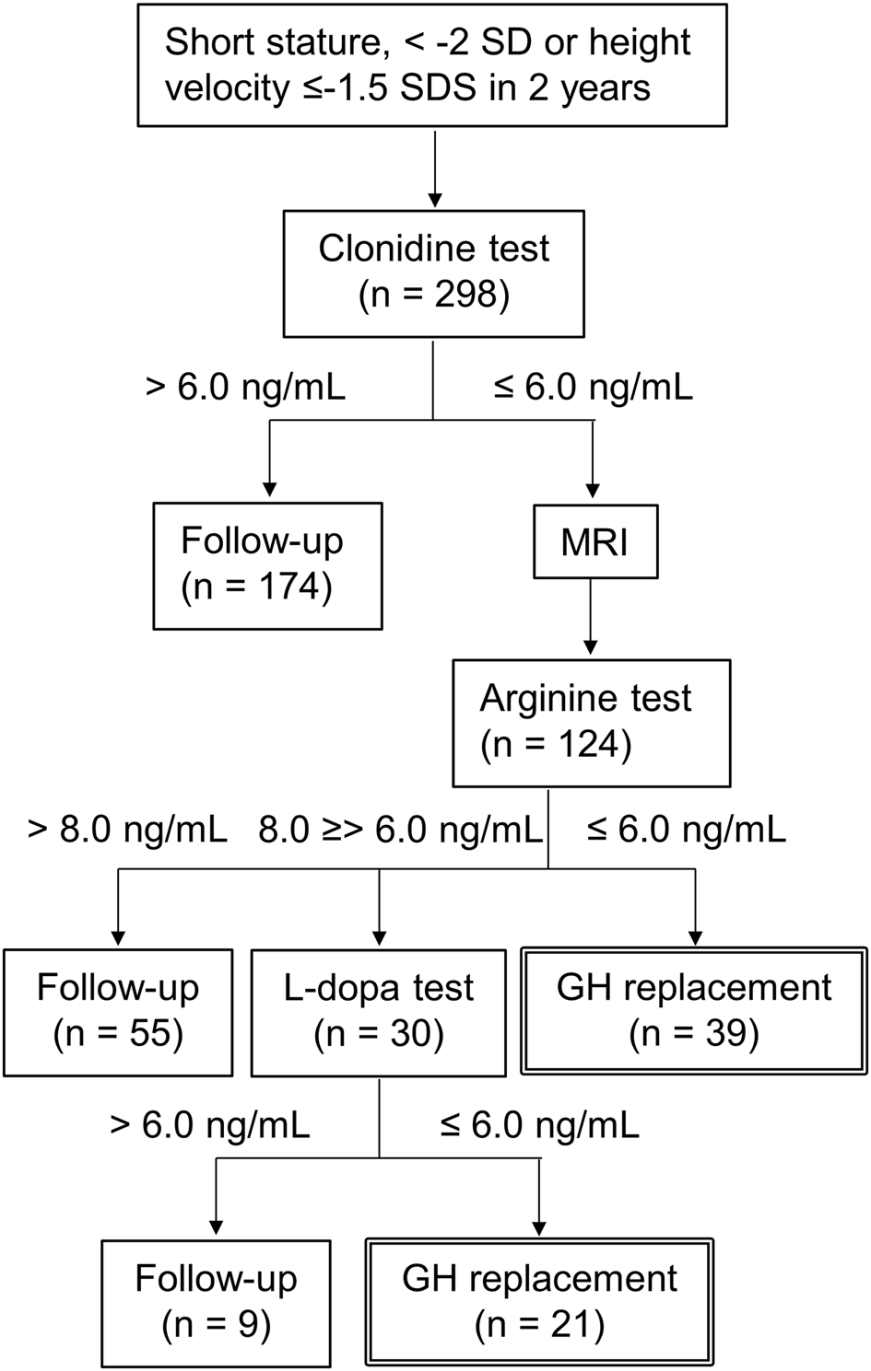
Algorithm of the stimulation tests using clonidine, arginine, and L-dopa. If peak growth hormone (GH) in both clonidine and arginine tests was ≤ 6.0 ng/mL, GH replacement therapy was initiated. When the peak GH in arginine test was 6.0–8.0 ng/mL, the third stimulation test using L-dopa was performed.

General biochemical tests, thyroid function test, bone age, and IGF-1 were examined before GH secretion test in consecutive patients who met the inclusion criteria. The radius, ulna, and short bone method was used for evaluating bone age^11^. The patients were divided into GH and non-GH groups according to the response to the GH secretion test (GH, 60; non-GH, 238). In Japan, GHD is diagnosed when the peak GH is ≤ 6.0 ng/mL in two GH secretion tests^9^. A cutoff of 6 ng/mL was determined by the Japanese National Health Insurance program. Stimulation tests using clonidine, arginine, and L-dopa were performed in that order, using the algorithm shown in Fig. 1. GHD was diagnosed if the GH peak levels were ≤ 6 ng/mL in the two stimulation tests. If the GH peak was above the cutoff level in the clonidine stimulation test, the next stimulation test was not performed. If the GH peak of the arginine stimulation test was 6–8 ng/mL, the third L-dopa stimulation test was performed. If the GH peak of the arginine stimulation test was > 8 ng/mL, the third test was not performed as GHD was unlikely to be present. Glucagon was not used in this study because glucagon requires a long examination time of 180 min. Insulin was also not used in this study because of its potentially serious side effects and we were not accustomed to its use.

After overnight fasting, the stimulation test was started at 6:30 for children < 6 years old and at 9:00 for those > 6 years old because of fasting tolerance. Sampling was done at 0, 30, 60, 90, and 120 min. Clonidine (5 µg/kg), arginine (10 mg/kg), and L-dopa (10 mg/kg) were administered as the stimuli for the GH secretion test. Sex steroids were not used for priming before the GH secretion test. After the diagnosis of GHD, head MRI was performed before starting GH replacement therapy.

### Hormone assays

Serum IGF-1 was measured by electrochemiluminescence immunoassay (Elecsys IGF-1; Roche Diagnostics, Tokyo, Japan), which was calibrated against the WHO International Standard 02/254. The values of serum IGF-1 were transformed into SDs, according to the established reference ranges of the assay for sex and calendar age^12^. GH was measured by immunoenzymometric assay (E Test TOSOH II HGH; Tosoh Co., Ltd., Tokyo, Japan), which was standardized against the WHO International Standard 98/574. According to the manufacture's datasheet, the intra- and interassay coefficients of variation (CV) for IGF-1 was < 10% and < 20%, and those for GH was < 10% and < 15%. As GH was measured in the hospital, we tested for intra-assay CV for GH in our hospital and found that it was 2% on average. Interassay CV for GH in our hospital was not tested. IGF-1 was measured by the testing company.

### Statistical analysis

We calculated point estimates for IGF-1 (SD) sensitivity, specificity, positive predictive value (PPV), negative predictive value (NPV), diagnostic efficiency (DE), positive likelihood ratio (PLR), and negative likelihood ratio (NLR) for predicting the presence of GHD. Data were shown as median (interquartile range) for chronological and bone age, and as mean ± SD for the other numerical variables. To exclude the influence of IGF-1 levels that vary with age, the GHD and non-GHD groups were subclassified into two groups according to age: older than 6 years and younger than 6 years. Based on the f-test, Student’s t-test was performed in the case of homoscedasticity and the Mann–Whitney U test was performed in the case of unequal variances to compare the IGF-1 level and other variables between the two groups. Spearman’s rank correlation coefficient test was performed to investigate the relationship of IGF-1 (SD) with age, bone age, height (SD), target height (SD), height velocity before examination (SD), weight (SD), body mass index (BMI) (SD), and maximum peak GH (ng/mL). Correlation was defined as very weak if < 0.2, weak if ≥ 0.2 and < 0.4, moderate if ≥ 0.4 and < 0.6, strong if ≥ 0.6 and < 0.8, and very strong if ≥ 0.8. Receiver operating characteristic (ROC) analysis with the Youden index was used to compare the discriminatory performances of IGF-1 in the diagnosis of GHD. Based on the area under the ROC curve (AUC), performance was considered as acceptable if > 0.7 and ≤ 0.8 and excellent if > 0.8.

All statistical analyses were performed using EZR (Saitama Medical Center, Jichi Medical University, Saitama, Japan)^13^, which is a graphical user interface for R (The R Foundation for Statistical Computing, Vienna, Austria). More precisely, it is a modified version of R commander designed to add statistical functions frequently used in biostatistics.

### Ethics approval

The study was approved by the ethics committee of Aichi Medical University (originally 2015-H359 but revised to 2020-H041 as the study period was updated).

### Consent to participate and for publication

The parents of the study subjects provided consent to participate and for publication after full explanation of the purpose and nature of all the procedures used in this study.

## Results

The patients included in this study had a median age of 4.98 years (interquartile range, 3.21–9.38 years). We identified 60 children with GHD and 238 children without GHD (non-GHD) (Fig. 1), with male preponderance of 53.4%. Four patients were diagnosed with organic GHD because of inflammation or a tumor on MRI (lymphocytic hypophysitis, n = 2; craniopharyngioma, n = 1; and cerebral myeloma, n = 1). In two patients, empty sella was detected on MRI, but this finding was considered a normal variation. The MRI findings were unremarkable in all the other patients. The backgrounds of these patients are described in Table 1. Height (SD), target height (SD), and IGF-1 (SD) did not differ significantly between the GHD and non-GHD groups. The GHD group had a significantly higher post-examination height velocity, body weight (SD), and BMI (SD) but a lower maximum peak GH than the non-GHD group. Except for weight and BMI, the trends for all parameters in the ≤ 6 years and > 6 years age groups were the same as those for the entire cohort. Height, target height, and IGF-1 did not vary significantly between the GHD and non-GHD in both the ≤ 6 years and > 6 years age groups (Table 1).

**Table 1:** Patient characteristics.

	All patients			≤ 6 years			> 6 years		
	GHD group	Non-GHD group	p value	GHD group	Non-GHD group	p value	GHD group	Non-GHD group	p value
	(n = 60)	(n = 238)		(n = 34)	(n = 135)		(n = 26)	(n = 103)	
Male:female ratio	34:26	125:113	0.664	18:16	70:65	1	16:10	55:48	0.513
Age (years)	5.10 (3.37–10.03)	4.94 (3.19–9.17)	0.565	3.37 (3.00–3.85)	3.19 (2.39–3.71)	0.209	10.45 (8.88–11.35)	9.51 (8.18–11.64)	0.493
Bone age (years)	3.21 (2.42–7.90)	3.21 (2.42–7.50)	0.778	2.42 (2.25–3.00)	2.50 (1.67–2.94)	0.834	8.33 (7.02–10.40)	7.83 (6.69–10.40)	0.683
Prepubertal: pubertal	58:2	224:14	0.748	34:0	135:0	1	24:2	89:14	0.524
Height (SD)	− 2.32 ± 0.76	− 2.37 ± 0.82	0.645	− 3.30 ± 2.90	− 3.38 ± 2.15	0.86	− 0.33 ± 5.20	− 0.28 ± 4.53	0.962
Target height (SD)	− 0.49 ± 0.69	0.47 ± 0.71	0.869	− 0.59 ± 0.75	− 0.42 ± 0.68	0.218	− 0.37 ± 0.59	− 0.54 ± 0.75	0.285
**Height velocity (SD)**									
Before examination	− 1.39 ± 2.35	− 0.97 ± 1.93	0.157	− 1.25 ± 2.15	− 1.23 ± 1.52	0.962	− 1.57 ± 2.62	− 0.63 ± 2.32	0.077
After examination	2.60 ± 3.10	− 0.12 ± 2.96	< 0.001	2.59 ± 2.77	− 0.25 ± 2.44	< 0.001	2.60 ± 3.54	0.09 ± 3.62	< 0.01
Weight (SD)	− 1.61 ± 0.96	− 2.00 ± 1.08	0.0144	− 2.87 ± 3.44	− 3.18 ± 3.67	0.652	− 0.71 ± 3.05	− 1.06 ± 2.72	0.570
BMI (SD)	− 0.11 ± 1.11	− 0.50 ± 1.12	0.0207	0.01 ± 1.14	− 0.16 ± 1.12	0.435	− 0.29 ± 1.29	− 0.87 ± 1.06	0.019
Max. Peak GH (ng/mL)	5.94 ± 2.63	11.17 ± 3.97	< 0.001	6.25 ± 2.85	10.61 ± 3.34	< 0.001	5.54 ± 2.31	11.92 ± 4.58	< 0.001
IGF-1 (SD)	− 1.43 ± 1.14	− 1.12 ± 0.88	0.23	− 1.12 ± 0.67	− 0.90 ± 0.74	0.12	− 1.79 ± 1.48	− 1.42 ± 0.96	0.126

The values are expressed as medians (IQR) or means ± SD.

SD, standard deviation; BMI, body mass index; GH, growth hormone; GHD, growth hormone deficiency; IGF-1, insulin-like growth factor; IQR, interquartile range.

**Figure 2:**
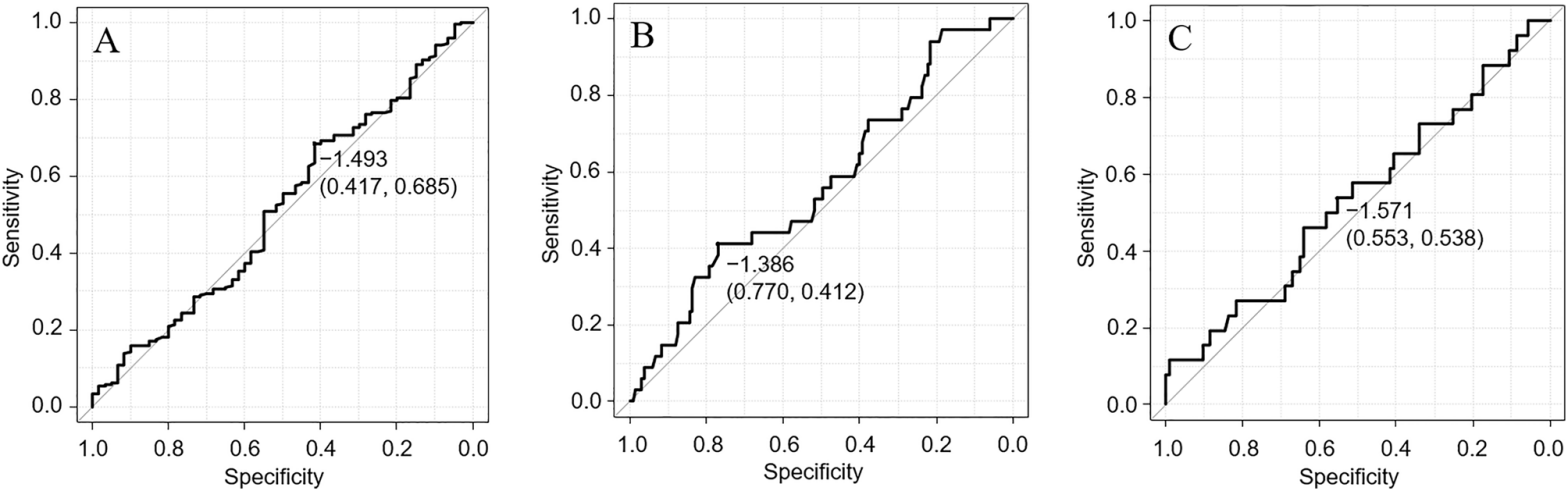
Receiver operating characteristic (ROC) curve of IGF-1 (SD) for the diagnosis of growth hormone deficiency (GHD) for all patients (A), patients aged ≤ 6 years (B), and patients aged > 6 years (C). ROC analysis for all patients showed the best diagnostic accuracy at an IGF-1 cutoff of − 1.493 standard deviation (sensitivity, 0.685; specificity, 0.417; and area under the ROC curve, 0.517).

ROC analysis showed the best diagnostic accuracy at an IGF-1 cutoff of − 1.493 SD (sensitivity, 0.685; specificity, 0.417; PPV, 0.25; NPV, 0.823; DE, 0.631; PLR, 1.322; NLR, 0.852; AUC = 0.517) (Fig. 2A). Thus, using the IGF-1 (SD) cutoff of − 1.493, a correct diagnosis was possible in 26 patients with GHD and 161 subjects without GHD. ROC analysis with stratification by age revealed that the AUC for ≤ 6 years and > 6 years groups was 0.57 (Fig. 2B) and 0.536 (Fig. 2C), respectively.

IGF-1 (SD) across the GHD and non-GHD groups showed normality in the Kolmogorov–Smirnov test but not in the Shapiro–Wilk test, and outliers were in the IGF-1 (SD) phase. Because of the possible significant impact on the number of relationships, we decided to analyze the IGF-1 (SD) using Spearman’s rank correlation coefficient. The correlation of IGF-1 (SD) was weak with age (r =  − 0.264, p < 0.001), bone age (r =  − 0.26, p < 0.001), height velocity before examination (SD) (r = 0.22, p < 0.001), weight (SD) (r = 0.219, p < 0.001), and BMI (SD) (r = 0.241, p < 0.001) and very weak with height (SD) (r = 0.0815, p = 0.16), target height (SD) (r =  − 0.05, p = 0.393), and maximum GH peak (r = 0.129, p = 0.0257).

To clarify the relationship between pretreatment IGF-1 and response to GH, we compared the height velocity (SD) between groups with IGF-1 above (n = 34) and below (n = 26) the cutoff value (− 1.493 SD). Pre- and posttreatment height velocity (SD) were similar between the groups (pretreatment, − 1.28 ± 2.25 vs. − 1.52 ± 2.51, p = 0.696; posttreatment, 2.48 ± 3.15 vs. 2.74 ± 3.09, p = 0.755).

To assess the efficacy of the third stimulation test, patients diagnosed with GHD on the second (n = 39) and third tests (n = 21) were compared (Table 2). Pre- and posttreatment growth velocities (SD) were similar between these groups (pretreatment, − 1.66 ± 2.36 vs. − 0.89 ± 2.31, p = 0.232; posttreatment, 2.44 ± 3.00 vs. 2.87 ± 3.33, p = 0.618). To be more precise, the height velocity was compared according to age and sex groups (Table 2). The age was classified into two categories as those aged ≤ 9 years and those aged > 9 years in boys as well as those aged ≤ 8 years and those aged > 8 years in girls. In any subgroup, height velocity before and after the examination was not significantly different between those diagnosed on two and three tests (Table 2).

**Table 2:** Analysis of height velocity according to the age group before and after examination.

	HV before examination			HV after examination		
	2 tests	3 tests	p value	2 tests	3 tests	p value
All patients with GHD	− 1.66 ± 2.36 (n = 39)	− 0.89 ± 2.31 (n = 21)	0.232	2.44 ± 3.00	2.87 ± 3.33	0.618
Male with GHD	− 1.69 ± 2.57 (n = 22)	− 1.03 ± 2.72 (n = 12)	0.49	2.18 ± 3.31	2.94 ± 3.02	0.523
Male with GHD, ≤ 9 years old	− 1.47 ± 2.11 (n = 12)	− 1.26 ± 2.68 (n = 9)	0.845	2.44 ± 2.76	2.58 ± 3.24	0.914
Male with GHD, > 9 years old	− 1.96 ± 3.14 (n = 10)	− 0.35 ± 3.33 (n = 3)	0.458	1.88 ± 4.03	4.01 ± 2.44	0.414
Female with GHD	− 1.61 ± 2.12 (n = 17)	− 0.70 ± 1.75 (n = 9)	0.281	2.75 ± 2.63	2.77 ± 3.89	0.99
Female with GHD, ≤ 8 years old	− 1.38 ± 2.16 (n = 13)	− 1.01 ± 1.62 (n = 7)	0.698	3.16 ± 2.75	2.04 ± 1.64	0.343
Female with GHD, > 8 years old	− 2.34 ± 2.10 (n = 4)	0.40 ± 2.35 (n = 2)	0.216	1.54 ± 2.06	5.33 ± 9.38	0.431

HV, height velocity; GHD, growth hormone deficiency.

## Discussion

We found that IGF-1 had poor accuracy as demonstrated by low AUC, and poor sensitivity, specificity, and DE for the best cutoff of − 1.493 SD. Furthermore, age stratification did not improve the accuracy of IGF-1. The correlation analysis revealed that none of the items increased the diagnostic power of IGF-1 for GHD screening.

IGF-1 has been reported to be useful in the screening of GHD in some studies^3–7^ but not in others^1,8^. The reason for these contradictory results is that the patient groups and GHD cutoff values differed between these studies. First, the inclusion criteria may create differences in patient backgrounds. In previous studies on the usefulness of IGF-1 for GHD screening, patients were selected according to bone age^2^, target height^3,6^, or catch-up growth^3^ in addition to short stature and/or height velocity. These variations in inclusion criteria might superficially improve the sensitivity and specificity of IGF-1. Second, different GH cutoff levels for GHD were selected: ≤ 5 ng/mL^5,6^, ≤ 6 ng/mL^9^, ≤ 7 ng/mL^1^, ≤ 8 ng/mL^3,8^, and ≤ 10 ng/mL^4,14^. In the case of ≤ 8^8^ or ≤ 10 ng/mL^14^, the prevalence of GHD in patients with short stature was 29%–34%, which was higher than that in our study (20.1%). Since disease prevalence affects sensitivity, specificity, PPV, and NPV, IGF-1 is not a useful screening test in a patient population with low prevalence of GHD. In the cohort of this study, the prevalence of GHD was decreased to 12.8% when the GH cutoff level of 5 ng/mL was selected. Therefore, when evaluating the efficacy of IGF-1, comparisons should be made at the same GH cutoff levels.

Bone age, target height, and height velocity should be taken into consideration before selecting patients for the GH secretion test^2^. In our study, bone age, target height, and height velocity before the examination were similar between the GHD and non-GHD groups. Even after combining these conditions with IGF-1, the diagnostic power of IGF-1 for GHD screening did not increase. Therefore, it would be difficult to distinguish patients with GHD from those without GHD using those parameters.

To clarify the relationship between pretreatment IGF-1 and response to GH, height velocity (SD) between groups with IGF-1 above (n = 34) and below (n = 26) the cutoff value (− 1.493 SD) was compared. Pre- and posttreatment height velocity (SD) were similar between the groups. IGF-1 was reported to be weakly correlated with the clinical endpoints of GH treatment^15^. Therefore, it would be difficult to predict the degree of improvement prior to GH treatment using pretreatment IGF-1.

We performed the third stimulation test when the results of GH secretion in the first and second tests were different. Although a sufficient GH response in one stimulation test rules out GHD in most cases^2^, the utilization and interpretation of the drugs used in the stimulation test depends on the facility^1,16^. In this study, pre- and posttreatment growth velocities were similar between the patients diagnosed with GHD on the second and third tests (Table 2). In any subgroup, height velocity before and after examination was not significantly different between those diagnosed on two and three tests. This result indicated that patients diagnosed with GHD by the third test have the similar response to growth hormone as those diagnosed by the traditional method. Therefore, the third simulation test may have some significance in diagnosing patients with GHD.

The number of patients with GHD is low if consider their age range as between 3 and 9 years. Other features, such as cutoff values for GHD or obesity, may need to be evaluated in the present cohort. A study on younger children with GHD showed that 29% of the patients with short stature had GHD^14^. The cutoff value for GHD used in the said study was 10 ng/mL. If the cutoff value of 6 ng/mL used in our study was applied to that cohort, the number of patients with GHD would be lower. In addition, the GHD and non-GHD groups of that and our study had similar BMI. Therefore, the cause of the lower number of patients with GHD in our study may be owing to the lower cutoff value for GHD and not due to obesity.

Of further interest would be the fact that the patients with GHD in our study did not have low IGF-1 levels. The reason for this could be their nutritional status. In our cohort, other than height velocity after examination and maximum peak GH, only weight (SD) and BMI (SD) varied significantly between the GH and non-GHD groups. Some studies have reported a positive correlation between IGF-1 levels and BMI^17,18^. Therefore, the higher BMI (SD) in the GHD group than that in the non-GHD group could have contributed to the similar IGF-1 levels between the groups.

This study had several limitations. First, immunoassay for IGF-1 analysis is not the most sensitive assay. The variations in immunoassays used in different studies may result in variations in the reported efficacy of IGF-1. More accurate assays, such as LC–MS, may reveal the actual usefulness of IGF-1 for GHD screening. Second, the use of a third stimulation test is not a common practice. If one of the tests is normal, there is no need for a third one. Thus, if the cutoff for a normal GH peak is set at 6 ng/ml, all responses above 6 ng/ml should be considered normal. However, depending on the order of each stimulation test, the diagnosis of GHD may vary among patients. For example, a patient with a peak GH < 6 ng/mL in A and B stimulation tests and ≥ 6 ng/mL in C stimulation test would not be diagnosed with GHD if the order of the stimulation tests were A, C, and B. There is no evidence on the order of stimulation tests, and the order varies from institution to institution. In the present study, the response to GH was similar in patients who had substandard results in two of the two stimulation tests and in those who had substandard results in two of the three stimulation tests. Therefore, it is necessary to accumulate such cases to clarify the significance of the third stimulation test.

In conclusion, IGF-1 level had poor diagnostic accuracy as a screening test for GHD. Correlation analysis revealed that none of the items increased the diagnostic power of IGF-1. Therefore, IGF-1 should not be used alone for the screening of GHD. A predictive biomarker for GHD should be developed in the future.

## Data Availability

The data that support the findings of this study are available upon request from the corresponding author. The data are not publicly available because of privacy and ethical restrictions.
